# Comparison of the SenSmart™ and the INVOS™ neonatal cerebral near-infrared spectrometry devices

**DOI:** 10.3389/fped.2023.1243977

**Published:** 2023-08-25

**Authors:** Wariphan Wirayannawat, Jutharat Amawat, Nattaya Yamsiri, Bosco Paes, Ratchada Kitsommart

**Affiliations:** ^1^ Division of Neonatology, Department of Pediatrics, Faculty of Medicine Siriraj Hospital, Mahidol University, Bangkok, Thailand; ^2^ Nursing Division, Department of Pediatrics, Faculty of Medicine Siriraj Hospital, Mahidol University, Bangkok, Thailand; ^3^ Division of Neonatology, Department of Pediatrics, McMaster University, Hamilton, ON, Canada

**Keywords:** cerebral oxygen, INVOS, NIRS, SenSmart, tissue oxygenation

## Abstract

**Objectives:**

To determine the correlation and agreement between the SenSmart™ and the INVOS™ devices of neonatal cerebral regional oxygen saturation (CrSO_2_) measurements using neonatal sensors. The secondary objective was to develop a regression model that predicts CrSO_2_-INVOS values using CrSO_2_-SenSmart indices and determine whether the values between the devices are interchangeable.

**Methods:**

A prospective, cross-sectional study was conducted in infants during the first 4 weeks of life. Simultaneous, bilateral CrSO_2_ was measured using the SenSmart™X100 (CrSO_2_-SenSmart) or INVOS™ 5100C (CrSO_2_-INVOS) device in each frontoparietal area for 2 h. Five-minute CrSO_2_ values were extracted for analysis.

**Results:**

Thirty infants were recruited with 720 pairwise measurements and 26 (84%) were evaluated in the first week of life. Mean gestational age of the preterm and term infants was [30.9 ± 2.8 (*n* = 14) and 38.8 ± 1.1 (*n* = 16)] weeks, respectively. Overall CrSO_2_- was 77.08 ± 9.70% and 71.45 ± 12.74% for the SenSmart and INVOS, respectively (*p* < 0.001). The correlation coefficient (*r*) between the CrSO_2_-SenSmart and INVOS was 0.20 (*p* < 0.001). The mean difference between the CrSO_2_-SenSmart and INVOS was 5.63 ± 13.87% with −21.6% to 32.8% limits of agreement. The *r* and mean difference was 0.39 (*p* < 0.001) and 8.87 ± 12.58% in preterm infants, and 0.06 (*p* = 0.27) and 2.79 ± 14.34 in term infants.

**Conclusion:**

The CrSO_2_-SenSmart tended to read higher than the CrSO_2_-INVOS device. There was no correlation between the CrSO_2_-SenSmart and the CrSO_2_-INVOS in term infants and it was weak in preterms. Due to imprecise agreement, the CrSO_2_-SenSmart values are not interchangeable with those of the CrSO_2_-INVOS.

## Introduction

Near-infrared spectroscopy (NIRS) is an additional non-invasive bedside device to evaluate regional tissue oxygenation. NIRS when combined with other monitoring techniques holds promise for the improved management and outcomes of critically ill neonates, especially extremely preterm infants (1–5), those with hemodynamic instability (6), encephalopathy (7–9), and cardiac disorders (10, 11). NIRS is most commonly utilized to measure cerebral regional oxygen saturation (CrSO_2_) to gain better understanding of cerebral oxygenation and autoregulation (12).

Commercial NIRS devices employ spatial resolved spectroscopy via a sensor that emits light at a wavelength in the near-infrared spectrum (700–900 nm). Although there are several commercial devices available for use in neonates, each one has specific characteristics including emitter wavelengths, distance between emitter and detectors, artery-to-venous (A:V) ratio, and an operational algorithm (13). This poses a challenge regarding indices generated across devices which impacts clinical interpretation and how best to employ different devices in the same patients (14–16).

The INVOS™ 5100C neonatal device employs the OxyAlert™ sensor that utilizes a single light emitting diode (LED) source that emits wavelengths at 730 and 810 nm and two detectors located 30 and 40 mm from the emitter. The spatial resolved spectroscopy technique excludes light absorption by hair or skull and solely monitors intracranial components. The device integrates the value of absorbed and scattered light in a specific algorithm with an estimated artery-to-venous ratio (AVR) of 25:75 (17).

The SenSmart™ X-100 (Nonin Medical Inc., Plymouth, MN) is another commercial NIRS device that monitors tissue oxygenation via the Equanox™ sensor and is adapted for use in neonates. Two LEDs emit 4 wavelengths of 730, 760, 810 and 880 nm and the 2 detectors are located 12.5 and 25 mm from each emitter, allowing 2 separate sets of a single emission with 2 detectors. Given dual emitters and detector sensor topology, the sensor generates 4 light paths which exclude extracranial contamination providing accurate tissue oxygenation (17). The algorithm AVR is 30:70. The sensor is durable which allows longer use that leads to better cost-effectiveness. However, studies of normative values and the clinical application of the SenSmart™ in neonates is limited.

Hyttel-Sorensen et al. (14) published an *in vitro* study showing good correlation of the 3 sensors utilized in the INVOS™ and NIRO™ technologies. Similarly in an *in vitro* study, Kleiser et al. (18) developed a predictive model involving various types and sizes of the sensors and oximeters that included INVOS™ and SenSmart™, but this was not validated in a clinical study. Andresen et al. (16) published a regression formula to predict the values of the SenSmart™ from measurements by the INVOS™. Dix et al. (15) published a moderate to good correlation of CrSO_2_ values across 3 different NIRS devices without a direct comparison between the SenSmart™ and the INVOS™.

Our primary objective was to explore the correlation between CrSO_2_ values measured by the SenSmart™ X-100 device that employs the neonatal/pediatric Equanox™ Advance 8004CB NA sensor (CrSO_2_-SenSmart) and the INVOS™5100C device that employs the neonatal OxyAlert™ sensor (CrSO_2_-INVOS). We hypothesized that both devices would have a strong correlation. The secondary objective was to develop a regression model that predicts CrSO_2_-INVOS using CrSO_2_-SenSmart indices.

## Material and methods

This prospective cross-sectional study was conducted in the neonatal intensive care unit (NICU), Siriraj Hospital in Bangkok, Thailand. We generally monitor CrSO_2_ in extremely low birthweight (ELBW) infants (birthweight; BW <1,000 g) early in the neonatal course, infants with hypoxic-ischemic encephalopathy (HIE), and those who have severe respiratory compromise. Eligible criteria included infants who were admitted to the NICU within the first 4 weeks of life and were allocated to CrSO_2_ by the responsible physician. Infants who had skin breakdown, inflammation, or birthmarks at the site of sensor placement were excluded. Subjects were enrolled only once in the study. Parental written consent was obtained prior to infant recruitment. The study protocol was approved by the institutional research committee.

Infants were placed supine and were undisturbed throughout the study period. Measurements of CrSO_2_ were performed with the INVOS™ 5100C device and OxyAlert™ sensor and the SenSmart™ X-100 device with neonatal/pediatric Equanox™ Advance 8004CB NA sensor. For skin protection in ELBW infants, the cover was not removed. Wijbenga et al. (3), reported similar CrSO_2_ values measured at the left and right frontoparietal area. We, therefore, compared the CrSO_2_ values between the two devices by simultaneously placing the sensor of each device on the forehead in the right and left frontoparietal position (19). The Equanox™ and OxyAlert™ sensors were first placed on the right and left frontoparietal areas, respectively and were wrapped with Easifix™ cohesive bandage around the head to ensure fixation. Both devices were synchronized for time and simultaneously monitored. After 1-hour, each sensor was transposed to the alternate site and monitoring continued for a further 1 h. Data from both devices were exported and averaged for 5-second values. The SenSmart™ X-100 analyzes CrSO_2_ every 1.5 s while the INVOS™ 5100C depicts the average 5-second value. Since variation of CrSO_2_ is common in the neonatal population (20, 21), we chose the value of the last 5-second average of each 5-minute epoch of the CrSO_2_-INVOS and the correspondent 5-second average of CrSO_2_-SenSmart values to permit simultaneous and independent comparisons. In view of the fact that the INVOS™ 5100C only displays values between 15% and 95%, values only within this range were included. Time points with poor signal quality as indicated by error codes were excluded.

### Statistical analysis

The sample size was derived from a previous clinical study that showed a correlation coefficient (*r*) of 0.5 between the CrSO_2_-INVOS and CrSO_2_-SenSmart (16). We hypothesized that the two devices would have a good positive correlation (*r* = 0.8) and estimated that 30 comparisons would be required.

Infant demographic characteristics are presented in number (percentage), mean (±standard deviation; SD) or median [25th percentile, 75th percentile; P25, P75] where appropriate. CrSO_2_ (%) values are presented in mean ± standard deviation (SD) and compared by independent *t*-test. We used Spearman correlation coefficient (*r*) to explore the association between CrSO_2_- SenSmart and CrSO_2_-INVOS and simple linear regression analysis to generate a regression model to predict CrSO_2_-INVOS values using CrSO_2_-SenSmart indices. Since the operational threshold of 55% has been generally used in neonatal practice (22, 23), we further analyzed the correlation and agreement between both devices based on the CrSO_2_-INVOS values <55% and ≥55%. Bland–Altman analysis was used to identify agreement between both devices and between predicted CrSO_2_-INVOS from Kleiser et al. [CrSO_2_-INVOS = 2.67 (CrSO_2_-SenSmart) −113.8%] (18) to our measured CrSO_2_-INVOS. Agreements are presented in mean differences and limits of agreement (bias ± 1.96 SD). Degree of bias was explored for the proportional value of CrSO_2_ by using Pearson correlational coefficient. IBM SPSS statistics 27 software was used for all analyses. A *p*-value less than 0.05 was considered statistically significant.

## Results

From January 1st, 2022, to January 1st, 2023, 30 infants were included in the study. None of the CrSO_2_-SenSmart measurements were higher than 95%. Therefore, all 720 pairwise readings were included for analysis. Demographic characteristics are shown in Table 1. Twenty-six infants (84%) were monitored during the first week of life. Fourteen (45.2%) and 16 (54.8%) were preterm and term infants with a mean gestational age (GA) and birthweight (BW) of 30.9 ± 2.8 weeks, 1,514.6 ± 590.4 g, and 38.8 ± 1.1 weeks, and 2,898.1 ± 511.1 g, respectively. The use of respiratory support was similar in preterm and term infants. Fourteen infants (46.7%) received inotropic drugs during the study period. The mean hemoglobin level of the preterm infants was significantly lower than term infants (12.2 ± 2.5 and 15.2 ± 2.3 g/dl, *p *= 0.002).

**Table 1:** Infant demographic characteristics and interventions during the study period.

	All	GA <37 weeks	GA ≥37 weeks	*p*-value
	(*N* = 30)	(*n* = 14)	(*n* = 16)	
Gestational age (weeks)	35.1 ± 4.5	30.9 ± 2.8	38.8 ± 1.1	<0.001*
Birth weight (g)	2,252.5 ± 885.5	1,514.6 ± 590.4	2,898.1 ± 511.1	<0.001*
Male sex	18 (60.0)	7 (50.0)	11 (68.8)	0.30
Postnatal age (hours)	60 [24, 144]	108 [24, 168]	46 [25, 90]	0.25
Respiratory support				
None	3 (10)	0 (0)	3 (18.8)	0.23
Oxygen cannula or hood	1 (3.3)	0 (0)	1 (6.3)	1.0
Non-invasive ventilation	4 (13.3)	3 (21.4)	1 (6.3)	0.32
Mechanical ventilation	17 (56.7)	7 (50.0)	10 (62.5)	0.49
High-frequency ventilation	5 (16.7)	4 (28.6)	1 (6.3)	0.16
Inotropic support	14 (46.7)	8 (57.1)	6 (37.5)	0.28
Hemoglobin (g/dl)	13.8 ± 2.8	12.2 ± 2.5	15.2 ± 2.3	0.002*

Data are presented as mean ± standard deviation, number (%) or median [25th percentile, 75th percentile]. GA; gestational age.

*p*-values compared differences between term and preterm infants, *p < 0.05 is statistically significant.

**Figure 1:**
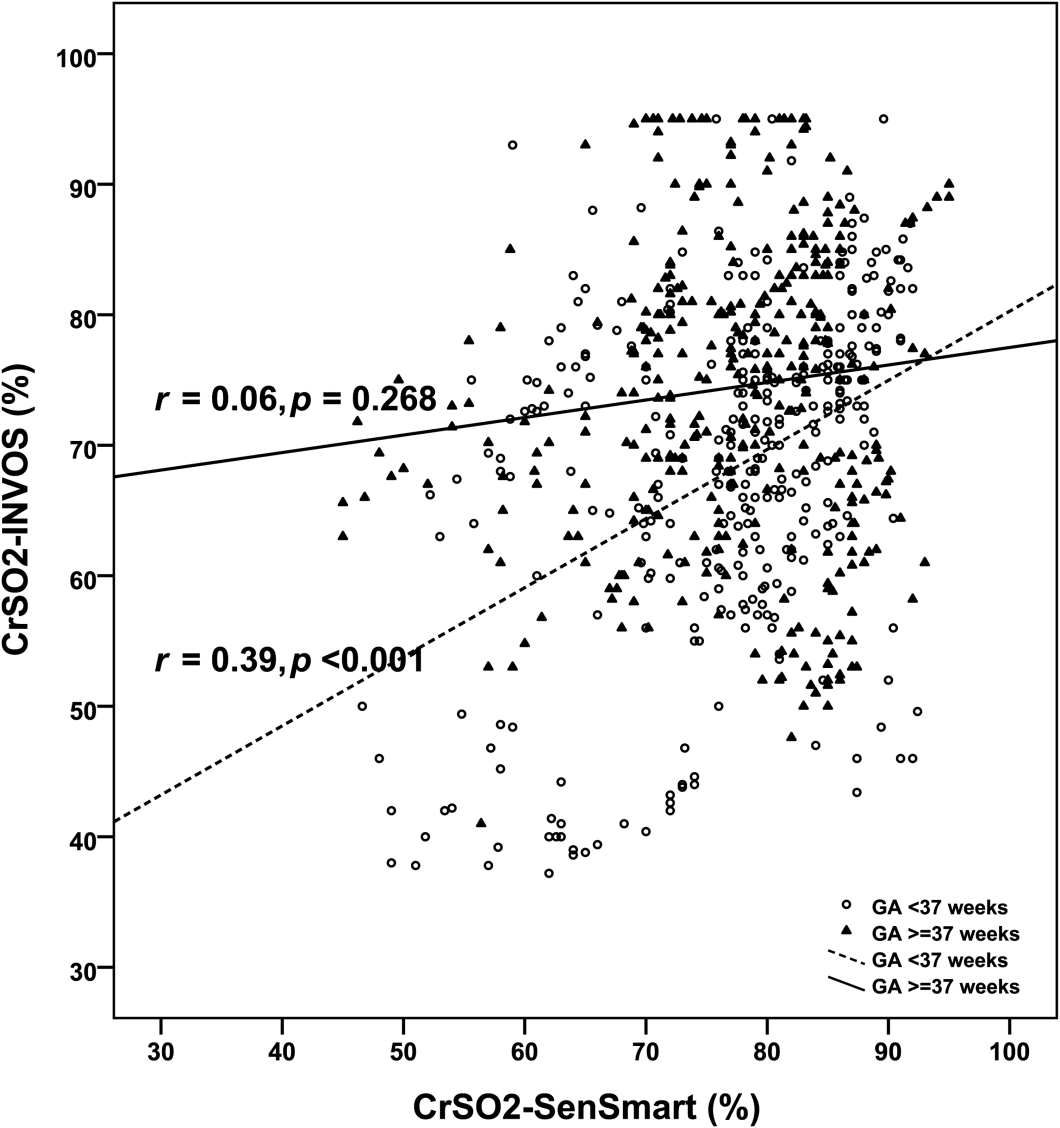
Scatter plots of cerebral regional oxygenation measurements based on the SenSmart™ X100 and the INVOS™ devices (*N* = 30). The dashed line demonstrates the regression line for preterm infants and the solid line demonstrates the regression line for term infants. *r*; correlation coefficient.

**Figure 2:**
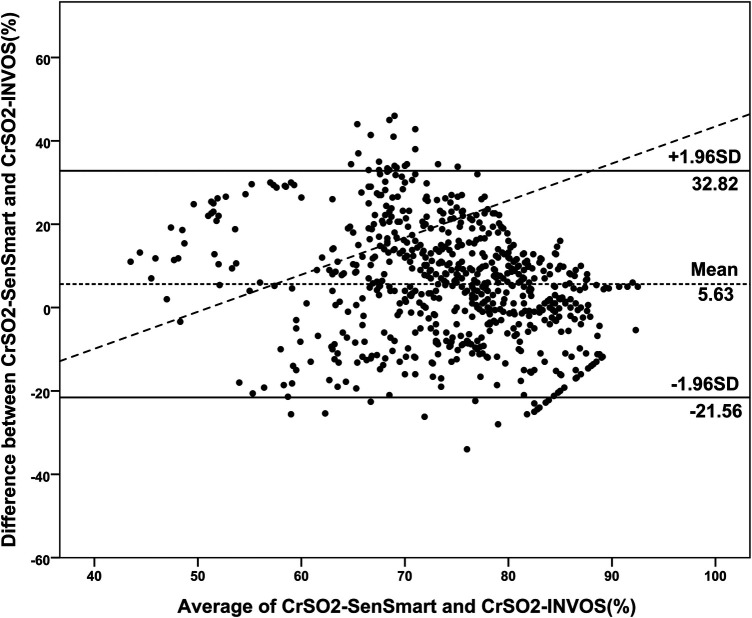
Bland–Altman plots of the differences in cerebral regional oxygenation values measured by the SenSmart™ X100 and the INVOS™ devices (*N* = 30). The dashed horizontal line indicates the mean of the differences between the two devices, and the solid horizontal lines indicate the upper and lower 95% limits of agreement.

Overall, the mean CrSO_2_-SenSmart (77.08 ± 9.70%) was significantly higher than the CrSO_2_-INVOS (71.45 ± 12.74%), *p *< 0.001. Figure 1 shows the scatter plot between CrSO_2_-SenSmart and CrSO_2_-INVOS. Linear regression analysis showed a low correlation between the two devices (*r* = 0.20, *p* < 0.001). The Bland–Altman plot (Figure 2) demonstrates the overall agreement between CrSO_2_-SenSmart and CrSO_2_-INVOS with a positive bias of 5.63 ± 13.87% and limits of agreement that ranged from −21.6% to 32.8%. However, the level of bias was proportional to the average degree of CrSO_2_-SenSmart and CrSO_2_-INVOS (*p *< 0.001).

The clinical diagnoses (*n*) among preterm infants were: Respiratory distress syndrome (4), hydrops fetalis (1), hypoxic-ischemic encephalopathy (HIE) (2), patent ductus arteriosus (1), multiple congenital anomalies (1), persistent pulmonary hypertension of the newborn (PPHN) (1), pulmonary atresia (2), and surgical conditions (2). The mean CrSO_2_-SenSmart (76.90 ± 10.10%) was significantly higher than the CrSO_2_-INVOS (68.04 ± 12.82%), *p* < 0.001 in preterm infants. There was a low correlation between the two devices (*r *= 0.39, *p* < 0.001). Bland–Altman analysis revealed a positive bias of 8.87 ± 12.58% with limits of agreement that ranged from −15.8% to 33.5%.

Among the 16 term infants, diagnoses (*n*) included: HIE with therapeutic hypothermia (7), traumatic brain injury (1), severe anemia with post-cardiac arrest (1), PPHN (2), septic shock (1), dextro-transposition of the great arteries with PPHN (1), tetralogy of fallot (1), gastroschisis (1), and achondroplasia with respiratory failure (1). The mean CrSO_2_-SenSmart (77.23 ± 9.34%) in term infants was also significantly higher than the CrSO_2_-INVOS (74.44 ± 11.91%) (*p* < 0.001). The correlation between the two devices was negligible (*r *= 0.06, *p* = 0.268). Bland–Altman analysis revealed a positive bias of 2.79 ± 14.34% with limits of agreement that ranged from −25.3% to 30.9%. Table 2 shows the agreement between the CrSO_2_-SenSmart and CrSO_2_-INVOS for each GA group. The CrSO_2_-SenSmart had a positive bias in all GA groups with wide limits of agreement.

**Table 2:** Correlation and agreement of cerebral regional oxygen saturation measured by the SenSmart™X-100 (CrSO_2_-SenSmart) and INVOS™ 5100C (CrSO_2_-INVOS) (*N* = 30).

	Correlation		Agreement	
	Correlation coefficient	*p*-value	Difference (%)	Limits of agreement (%)
All infants	0.20	<0.001*	5.63 ± 13.87	−21.6% to 32.8%
Gestational age (weeks)				
≥37 (*n* = 16)	0.06	0.268	2.79 ± 14.34	−25.3% to 30.9%
<37 (*n* = 14)	0.39	<0.001*	8.87 ± 12.58	−15.8% to 33.5%
34–36 (*n* = 3)	−0.26	0.028*	5.50 ± 11.09	−16.2% to 27.2%
28–33 (*n* = 9)	0.39	<0.001*	11.04 ± 13.14	−14.7% to 36.8%
<28 (*n* = 2)	−0.15	0.326	4.15 ± 9.51	−14.5% to 22.8%
CrSO_2_-INVOS				
<55%	0.53	<0.001*	25.17 ± 10.97	3.67% to 46.65%
≥55%	0.17	<0.001*	3.38 ± 12.34	−20.79% to 27.57%

Data are presented as mean ± standard deviation.

* *p*-value <0.05 is statistical significance.

Seventy-four pairwise comparisons (10.3%) had CrSO_2_-INVOS values <55% while the other 646 pairwise comparisons (89.7%) had CrSO_2_-INVOS ≥55%. Interestingly, there was a moderate positive correlation in the CrSO_2_-INVOS <55% (*r* = 0.53, *p *< 0.001) while the positive correlation was low in CrSO_2_-INVOS ≥55% (*r *= 0.17, *p* < 0.001).

During the study, 1 infant (3.3%) received dopamine, 11 (36.7%) received dobutamine, 7 (23.3%) received epinephrine, 9 (30%) received norepinephrine, 4 (13.3%) received milrinone, and 6 infants (20%) received hydrocortisone. In total, ten term (62.5%) and 6 preterm infants (42.9%) received inotropic support. We further explored the effect of hemodynamic status on agreement between the devices. Both devices had negligible correlation in infants who received inotropic support (*r *= 0.13, *p* = 0.009) and there was a low positive correlation among infants who did not receive inotropic agents (*r* = 0.31, *p *< 0.001).

Since term infants had a negligible correlation between the devices, we created regression model to predict CrSO_2_-INVOS values from CrSO_2_-SenSmart indices only for preterm infants which is shown in Table 3.

**Table 3:** Regression model of cerebral regional oxygen saturation measured by the SenSmart™X-100 (CrSO_2_-SenSmart) to predict the value equivalent to the INVOS™ 5100C (CrSO_2_-INVOS).

All infants (*N* = 30)	CrSO_2_-INVOS = 0.34 x CrSO_2_-SenSmart + 45.3% (95% CI 38.1–52.5)
GA <37 weeks (*n* = 14)	CrSO_2_-INVOS = 0.53 x CrSO_2_-SenSmart + 27.3% (95% CI 17.7–36.9)
With inotropic agents (*n* = 8)	CrSO_2_-INVOS = 0.54 x CrSO_2_-SenSmart + 28.8% (95% CI 11.7–45.8)
Without inotropic agents (*n* = 6)	CrSO_2_-INVOS = 0.49 x CrSO_2_-SenSmart + 29.1% (95% CI 17.1–41.0)

To validate the regression equation from Kleiser et al. (18) we compared predicted CrSO_2_-INVOS values from CrSO_2_-SenSmart measurements with our monitored CrSO_2_-INVOS indices. The correlation coefficient was 0.2 and the mean difference was 20.54 ± 25.74% with limits of agreement that ranged from −29.90% to 70.99%.

## Discussion

CrSO_2_ monitoring is more commonly used in the neonatal setting (24). Several studies demonstrate an association of CrSO_2_ with occurrences of certain morbidities such as necrotizing enterocolitis (25), respiratory insufficiency, intraventricular hemorrhage (26) and prediction of long-term outcomes (27). However, publications indicate a variation in the choice of NIRS devices and sensors. Currently, there are several devices and neonatal sensors available for use and there is little guidance whether measurements and trends noted in previous studies are uniformly applicable across devices (28).

Several factors need to be considered before accepting NIRS values in neonatal care. These include type of devices, type and size of the sensor, location of sensor placement, and patient's weight. While the INVOS™ 5100C was most commonly used in a large number of studies and generated an operational threshold for clinical intervention (23, 29), newer NIRS devices with updated LED wavelengths, design, and algorithms are now available to improve accuracy of the readings (28). Although values across devices are not interchangeable based on specific topography, they should correlate with each other to facilitate and interpret findings in clinical practice.

Our *in vivo* results supported by real-world evidence in the NICU, indicates that the CrSO_2_-SenSmart reads 5.75% higher than the CrSO_2_-INVOS (*p* < 0.001) device. The trend of higher readings with Equanox™ sensors compared to other sensors or devices align with the *in vitro* study (18). We postulate that the unique algorithm and higher AVR of the SenSmart™ contributes to the positive bias that we detected. Disappointingly, we found a low positive correlation between the two devices (*r* = 0.20) but, more importantly, the imprecision in the mean difference (SD 13.87%) and wider limits of agreement −21.6% to 32.8%) makes it more difficult to predict CrSO_2_-INVOS values accurately utilizing the SenSmart™ measurements. Although the maximum value of the CrSO_2_-SenSmart is 100%, compared to 95% in the CrSO_2_-INVOS, there were no CrSO_2_-SenSmart values higher than 95%. Therefore this did not impact our results. Interestingly, the correlation between both devices was better in preterm infants (*r* = 0.39, *p* < 0.001) which corresponds with the study by Andresen et al. (16) who found a moderate correlation between the INVOS™ and the SenSmart™ (*r*^2^ = 0.46) in preterm infants with apnea. The weaker correlation in term infants could be explained by the higher values detected in CrSO_2_-INVOS ≥55%), which could also contribute to the poor correlation between the devices. Moreover, the heterogenous disorders associated with fluctuating tissue oxygenation in term infants particularly hypoxemia, hypocarbia, and hemodynamic instability related to persistent pulmonary hypertension, severe anemia, or critical congenital heart disease and greater use of inotropic drugs may enhance measurement error across the devices (14, 30). The complexity of abnormal tissue oxygenation in the real-life situation also compliments our finding of the low correlation between our measured values and the predicted value from the *in vitro* study (18). However, the wide limits of agreement reported by Andresen et al. (16) and this study compromise the ability to accurately predict INVOS™ CrSO_2_ readings from the SenSmart™. Moreover, the regression model derived from the previous *in vitro* study (18) when applied to our study was also imprecise and cannot be applied in clinical settings.

We explored the potential of interchanging CrSO_2_ values between the two NIRS devices in sick neonates. The chosen average CrSO_2_ of the last 5 s of every 5-minute epoch made each value independent of each other. Along with a large number of comparisons, we analyzed the impact of gestational age on CrSO_2_ and the effect of inotropic agents. CrSO_2_ values are potentially interchangeable in preterms but not term infants. Nevertheless, the imprecise agreement jeopardizes accurate prediction of values in any gestational age. Inotropic agents employed to stabilize hemodynamic status could compromise tissue oxygenation and concomitantly have a vasoactive effect. This may have potentially led to a variation of values between the two devices over and above the variation resulting from individual device-related algorithms and A:V ratios. We found low correlations (*r* = 0.13 and 0.31) whether the subjects received or did not receive inotropic agents, respectively). Therefore, the CrSO_2_ values from both devices were not interchangeable even if using the predictive model. The operational threshold that was generated from the INVOS™ 5100C device using the *in vitro* model of Kleiser et al. (18) cannot be properly transferred to the SenSmart™ or other NIRS devices and is likely not valid in a clinical setting. CrSO_2_ must be monitored using the same device to justify the trend of changes since it is difficult to establish what a clinically relevant threshold should be for a device that is not INVOS™.

There are several limitations of this study that merit consideration. First, the negligible correlation in term infants could have been due to motion artifact (30). Since we compared only the values extracted from the devices, we could not account for the same in the recordings which are more likely to occur in active term infants. However, the large number of paired samples, should have minimized this confounder. Additionally, recognizing this potential limitation, we derived regression equations for only the overall cohort and preterm infants. Second, the number of pairwise comparisons were more than our baseline estimate. We selected our sample size based on a good correlation between devices from a previous study (16) but the same did not hold true in our findings. Therefore, a larger study with a predetermined sample size based on the low correlation from our findings is warranted to expand the generalizability of our results. Last, although the previous study revealed similar CrSO_2_ values between the left and right frontoparietal areas (19), we accepted that they were non-identical and perhaps contributed to the overall bias. However, this should not have affected the degree of correlation which in part was overcome by the large pairwise samples included in our analysis.

## Conclusion

The CrSO_2_-SenSmart device tends to read higher than the CrSO_2_-INVOS with a correspondingly low correlation, particularly in in term infants. Both the predictive models derived from a previous study and from this study, cannot accurately predict CrSO_2_-INVOS from CrSO_2_-SenSmart values, due to imprecise agreement. Therefore, the values of each device are not interchangeable. The type of device and sensor must be carefully considered when implementing CrSO_2_ values from previous studies into clinical practice.

## Data Availability

The raw data supporting the conclusions of this article will be made available by the authors, without undue reservation.
